# Clinical Characteristics of Cancer Patients With COVID-19: A Retrospective Multicentric Study in 19 Hospitals Within Hubei, China

**DOI:** 10.3389/fmed.2021.614057

**Published:** 2021-10-05

**Authors:** Deliang Guo, Haitao Wang, Qian Zhu, Yufeng Yuan

**Affiliations:** Department of Hepatobiliary and Pancreatic Surgery, Zhongnan Hospital of Wuhan University, Wuhan, China

**Keywords:** COVID-19, SARS-CoV-2, pneumonia, cancer, antitumor therapy

## Abstract

**Objective:** This study aimed to determine the association between prognosis of COVID-19 patients with and without cancer. Moreover, we compared the prognosis of cancer patients subjected to anti-tumor therapy with those who have not undergone anti-tumor therapy in the past 6 months.

**Methods and Results:** A total of 7,926 adult patients with COVID-19 were retrospectively enrolled in Hubei Province,China between December 31, 2019 and February 20, 2020. Two hundred and seventy seven cancer patients (cancer group, median age 64 [IQR 56–70] years; 50.90% male) and 7,649 non-cancer patients were identified (non-cancer group, median age 55 [IQR 42–64] years; 48.19% male). The mortality rate was lower in the non-cancer group compared to the cancer group (4.50 vs. 9.03%; *P* < 0.001). The duration between onset and admission shorter in the cancer group (Days, 9 [IQR 5–18]) compared to the non-cancer group (Days, 10; [IQR 6–19]; *P* = 0.036). ICU occupancy was higher in the cancer group (n[%], 30[10.83%]) than in the non-cancer group (n[%], 314[4.11%]). In reviewing the anti-tumor therapy, data from 277 selected cancer patients were obtained out of which 74 patients had undergone anti-tumor therapy (mean age 65 [IQR 51–67] years; 45.95% male), 203 had not undergone anti-tumor therapy (non-anti-tumor therapy group, mean age 63 [IQR 53–75] years; 49.75% male) in the past 6 months. The mortality rate for the anti-tumor therapy group and the non-anti-tumor therapy group was similar (9.46 vs. 8.87%; *P* = 0.879).

**Conclusion:** The mortality rate was higher in COVID-19 patients with cancer compared to those without cancer. Moreover, anti-tumor therapy in the past 6 months did not worsen the prognosis of cancer patients with COVID-19.

## Introduction

COVID-19 is currently a global pandemic. About 122,000 patients have been infected with COVID-19 in China with 4.63% death rate based on the data from the Chinese Center for Disease Control. There have been 207.17 million confirmed cases of COVID-19, including 4.36 million deaths on Aug 16th, 2021, reported to WHO (1). Previous studies focused on the general epidemiologic survey, clinical presentation, or prognosis of mild and severe pneumonia cases (2–7). However, limited studies exist on the epidemiology and prognosis in tumor patients infected with SARS-CoV-2 (8–11).

Herein, this study reports general epidemiologic survey, clinical presentation, and prognosis of a subgroup based on a multicentric observational outcome study from a large cohort of COVID-19 patients in Hubei province. A higher mortality rate was reported to be associated with cancer patients infected with SARS-CoV2. Of note, recent anti-tumor therapy does not jeopardize the prognosis of cancer patients with COVID-19. We hope that the findings from this study can provide insights to others (12) who are similarly confronted with the COVID-19 challenges that arise from cancer research.

## Methods

### Study Procedure and Patient Cohorts

This observational multicenter cohort study was performed in 19 tertiary hospitals in China. The admission of patients in the 19 hospitals were performed uniformly according to an executive order issued by the Chinese government (13). This study was approved by the Central Ethics Committees (Clinical Ethical Approval No. 2020010) and ratified by the Institutional Ethics Committees in each hospital without any alterations. Informed consent from patient was waived by the ethics committees from each hospital due to the emerging pandemics. COVID-19 diagnosis was determined through clinical manifestations, chest CT, or real-time RT-PCR according to WHO interim guidance and the New Coronavirus Pneumonia Prevention and Control Program (5th edition) published by the National Health Commission of China (14, 15). The inclusion criteria were patients diagnosed with COVID-19. The following exclusion criteria were used to determine the patient cohort: Age less than 18, incomplete medical records, without exact outcome (discharge or death). pregnancy, acute lethal organ injury (e.g., acute myocardial infarction, acute pulmonary embolism, or acute stroke), decompensated or end stage of chronic organ dysfunction (e.g., decompensated cirrhosis, decompensated chronic renal insufficiency, or severe congestive heart failure), acquired immune deficiency syndrome (AIDS), severe trauma (e.g., parenchymatous organ rupture, bleeding, fracture). Based on these criteria, a total of 7,926 COVID-19 patients admitted from December 31, 2019 to February 20, 2020 in 19 designated hospitals in Hubei Province, China were initially evaluated for inclusion. Exactly 277 patients having cancer or have a history of cancer were selected to represent the cancer group with COVID-19. The other 7,649 patients were enrolled in the non-cancer group.

Chest computerized tomography (CT) or throat-swab specimens were obtained from all patients upon admission. A team of physicians evaluated the severity of COVID-19. The demographics, clinical characteristics, medical history, laboratory tests, radiological reports, therapeutic intervention, and outcome data were obtained from the electronic medical records of the patients. The final date of follow up for determining the outcome was April 16th, 2020.

### Data Collection

The clinical end point was defined by death or recovery at the time of discharge from the hospital. Following data were collected including patient demographic information (age, gender), medical histories and underlying diseases [e.g., hypertension, diabetes mellitus, chronic obstructive pulmonary disease (COPD), chronic liver disease, chronic kidney disease], tumor type and location, prior treatment history (chemotherapy, radiation therapy, targeted therapy, surgery), physical examination findings and clinical manifestation (fever, cough, fatigue, dyspnea, and comorbidities), laboratory data [e.g., complete blood count, C-reactive protein (CRP), procalcitonin (PCT), hepatorenal function test, serum cardiac enzyme concentration], radiologic report data [unilateral or bilateral infiltrates was classified according to computed tomography (CT) scan of the chest], invasive or non-invasive therapeutic interventions [e.g., antibiotics, antivirals, Chinese patent medicine, vasoactive drugs, hormone therapy drugs, invasive or non-invasive ventilation, renal replacement therapy, extracorporeal membrane oxygenation (ECMO) therapy] and clinical outcomes [sepsis and septic shock, acute respiratory distress syndrome (ARDS), acute kidney injury (AKI), acute liver injury (ALI), acute myocardial injury (AMI), disseminated intravascular coagulation (DIC), ICU stay, clinical end point]. All identity information of patients was removed and recoded before data analysis. The database did not contain any patient identity or confidential information. Data were independently reviewed and confirmed by two experienced physicians to guarantee accuracy. When there was disagreement between the two examiners, a third physician was called in to give an opinion. Criteria definition is shown in Supplementary Table 4.

### Statistics

Continuous variables were presented as the mean±SD for normally distributed continuous variables while median and interquartile range (IQR) for non-normally distributed continuous variables. For all categorical variables, binary dummy variables and percentage (%) were introduced to represent the categorical values. Continuous variables were studied using the *T*-Test or Mann-Whitney *U*-test. Associations between categorical dependent variables and independent categorical variables were evaluated using Pearson's chi squared test or Yates's correction for continuity analysis. A difference was considered significant if the two-side *p*-value was less than 0.05. Statistical analyses were carried out using SPSS Statistics v23.0 (IBM, Armonk, NY, USA) and the statistical analysis package R-3.6.3 (R Foundation for Statistical Computing, Vienna, Austria).

## Results

### The Association Between Prognosis of COVID-19 Patients With and Without Cancer

To determine the prognosis difference between COVID-19 patients with and without cancer, the study cohort included 7,926 COVID-19 patients admitted to 19 hospitals in Hubei, China. Of the 7,926 patients, 277 participants with cancer were defined as cancer group [median age 64 (IQR 56–70) years; 50.90% male] and the leaving 7,649 were defined as the non-cancer group [median age 55 (IQR 42–64) years; 48.19% male]. Patient characteristics and comorbidities at admission are shown in Table 1.

**Table 1:** Clinical characteristics and Comorbidities of patients with COVID-19 in Non-cancer group and cancer group on admission.

**Parameters**	**Total population**	**Non-cancer group**	**Cancer group**	***P***
	***N* = 7,926**	***N* = 7,649**	***N* = 277**	
	**Median (range) or n (%)**	**Median (range) or n (%)**	**Median (range) or n (%)**	
**Clinical characteristics on admission**				
Symptom onset to admission, median (IQR), day	10 (6–19)	10 (6–19)	9 (5–18)	0.036^*^
Age, median (IQR), y	55 (43–64)	55 (42–64)	64 (56–70)	<0.001^**^
Male gender, n (%)	3,827 (48.28%)	3,686 (48.19%)	141 (50.90%)	0.375
Fever, n (%)	5,757 (72.63%)	5,539 (72.41%)	218 (78.70%)	0.021^*^
Cough, n (%)	5,038 (63.56%)	4,878 (63.77%)	160 (57.76%)	0.041^*^
Fatigue, n (%)	2,568 (32.40%)	2,486 (32.50%)	82 (29.60%)	0.311
Dyspnea, n (%)	1,297 (16.36%)	1,254 (16.39%)	43 (15.52%)	0.700
**Comorbidities on admission**				
COPD (%)	61 (0.76%)	60 (0.78%)	1 (0.36%)	0.658
Diabetes Mellitus (%)	867 (10.94%)	840 (10.98%)	27 (9.75%)	0.271
Hypertension (%)	1,964 (24.78%)	1,880 (24.58%)	84 (30.32%)	0.030^*^
Chronic liver disease, n (%)	142 (1.79%)	133 (1.74%)	9 (3.25%)	0.103
Chronic renal diseases, n (%)	15 (1.94%)	148 (1.93%)	6 (2.17%)	0.784

*P-values were generated by the comparison between Non-cancer group and Cancer group*,

* *P < 0.05*,

** *P < 0.01*.

The cancer group was characterized by older age, lower prevalence of cough and higher prevalence of fever at presentation compared to the non-cancer group. Besides, the duration between onset and admission was shorter in the cancer group than in the non-cancer group [days, 9 (IQR 5–18) vs. 10(IQR 6–19); *p* = 0.036] (Table 1). The thoracic CT findings revealed higher prevalence of pulmonary infection lesions in cancer group compared to the non-cancer group (Supplementary Table 1). Laboratory examination on admission indicated increased liver enzymes (AST increase 22.02 vs. 14.77%, *p* < 0.001), kidney function abnormalities (CREA increase 10.83 vs. 5.43%, *p* < 0.001) and increased C-reactive protein (38.27 vs. 29.45%, *p* = 0.002) and procalcitonin level (27.80 vs. 16.16% *p* < 0.001) in the cancer group compared to the non-cancer group (Supplementary Table 1).

Regarding the inpatients medical treatment, compared to the non-cancer group, we found that patients in the cancer group suffered more from sepsis and septic shock (7.22 vs. 1.78%; *p* < 0.001), ARDS (18.05 vs. 10.16%; *p* < 0.001), AKI (7.22 vs. 2.01%; *p* < 0.001), ALI (13.36 vs. 6.86%; *p* < 0.001), AMI (12.64 vs. 4.93%, *p* < 0.001), DIC (2.53 vs. 0.43%, *p* < 0.001; Table 2). Moreover, the cancer group experienced more antiviral drugs (74.73 vs. 67.67%; *p* = 0.013), antibiotics (71.84 vs. 59.17%; *p* < 0.001), vasoactive drugs (15.52 vs. 4.52%; *p* < 0.001), hormone therapy drugs (35.02 vs. 25.70%, *p* < 0.001), ICU treatment (10.83 vs. 4.11%, *p* < 0.001), non-invasive ventilation (9.75 vs. 5.87%, *p* = 0.008) and invasive ventilation (6.14 vs. 2.26%, *p* < 0.001) compared to the non-cancer group (Table 2). Clinical end point showed a higher mortality rate in the cancer group than the non-cancer group (9.03 vs. 4.50%, *p* < 0.001; Table 2).

**Table 2:** Treatment and Outcome of patients with COVID-19 in Non-cancer group and cancer group.

**Parameters**	**Total population**	**Non-cancer group**	**Cancer group**	***P***
	***N* = 7,926**	***N* = 7,649**	***N* = 277**	
	**Median (range) or n (%)**	**Median (range) or n (%)**	**Median (range) or n (%)**	
**Treatment**				
Antiviral drug, n (%)	5,383 (67.92%)	5,176 (67.67%)	207 (74.73%)	0.013^*^
Antibiotics, n (%)	4,725 (59.61%)	4,526 (59.17%)	199 (71.84%)	<0.001^**^
Traditional Chinese medicine, n (%)	6,268 (79.08%)	6,050 (79.10%)	218 (78.70%)	0.874
Antidiabetic drug, n (%)	1,028 (12.97%)	970 (12.68%)	58 (20.94%)	<0.001^**^
Vasoactive drug, n (%)	389 (4.91%)	346 (4.52%)	43 (15.52%)	<0.001^**^
Systemic corticosteroids, n (%)	2,063 (26.03%)	1,966 (25.70%)	97 (35.02%)	<0.001^**^
Immunoglobin, n (%)	1,554 (19.61%)	1,478 (19.32%)	76 (27.44%)	<0.001^**^
Nasal cannula oxygen inhalationd, n (%)	5,127 (64.69%)	4,903 (64.10%)	224 (80.87%)	<0.001^**^
Invasive ventilation, n (%)	190 (2.40%)	173 (2.26%)	17 (6.14%)	<0.001^**^
Noninvasive ventilation, n (%)	476 (6.01%)	449 (5.87%)	27 (9.75%)	0.008^**^
Renal replacement therapy, n (%)	76 (0.96%)	73 (0.95%)	3 (1.08%)	0.922
Extracorporeal membraneoxygenation, n (%)	22 (0.28%)	21 (0.27%)	1 (0.36%)	0.755
**Outcome**				
Sepsis and Septic shock, n (%)	156 (1.97%)	136 (1.78%)	20 (7.22%)	<0.001^**^
ARDS, n (%)	827 (10.43%)	777 (10.16%)	50 (18.05%)	<0.001^**^
Acute kidney injury, n (%)	174 (2.20%)	154 (2.01%)	20 (7.22%)	<0.001^**^
Acute liver injury, n (%)	562 (7.09%)	525 (6.86%)	37 (13.36%)	<0.001^**^
Acute myocardial injury, n (%)	412 (5.20%)	377 (4.93%)	35 (12.64%)	<0.001^**^
DIC, n (%)	40 (0.50%)	33 (0.43%)	7 (2.53%)	<0.001^**^
ICU stay, n (%)	344 (4.34%)	314 (4.11%)	30 (10.83%)	<0.001^**^
Mortality, n (%)	369 (4.66%)	344 (4.50%)	25 (9.03%)	<0.001^**^

*P-values were generated by the comparison between Non-cancer group and Cancer group*,

* *P < 0.05*,

** *P < 0.01*.

### Prognosis of Cancer Patients Subjected and Not Subjected to Anti-Tumor Therapy in the Past 6 Months

A total of 277 COVID-19 cancer patients were enrolled in this subgroup study cohort. The cancer types of patients and num. of patients underwent anti-tumor treatment are displayed in Supplementary Table 3. We analyzed cancers of different sites, anti-tumor treatment in the past 6 months did not increase the mortality rate Supplementary Table 3. Of note, 74 patients underwent anti-tumor therapy in the past 6 months and were defined as anti-tumor therapy group [median age 65 (IQR 51–67) years; 45.95% male] whereas, the other 203 patients were classified as the non-anti-tumor therapy group [median age 63 (IQR 53–75) years; 49.75% male]. Patient characteristics at admission are highlighted in Table 3. Comparison of the general data (age, sex, onset to admission time, medical histories and underlying diseases, physical exam findings and clinical manifestation, radiologic report data) showed no statistical difference between the non-antitumor therapy group and antitumor therapy (*p* > 0.05; Table 3 and Supplementary Table 2).

**Table 3:** Clinical characteristics and Comorbidities of cancer patients with COVID-19 in Non-antitumor group and Antitumor group on admission.

**Parameters**	**Total population**	**Non- antitumor group**	**Antitumor group**	***P***
	***N* = 277**	***N* = 203**	***N* = 74**	
	**Median (range) or n (%)**	**Median (range) or n (%)**	**Median (range) or n (%)**	
**Clinical characteristics on admission**				
Symptom onset to admission, median (IQR), day	9 (5–18)	9 (5–20)	9 (6–18)	0.990
Age, median (IQR), y	64 (56–70)	63 (53–75)	65 (51–67)	0.788
Male gender, n (%)	141 (50.9%)	101 (49.75%)	40 (45.95%)	0.526
Fever, n (%)	218 (78.70%)	159 (78.33%)	59 (79.73%)	0.801
Cough, n (%)	160 (57.76%)	118 (58.13%)	42 (56.76%)	0.838
Fatigue, n (%)	82 (29.60%)	66 (32.51%)	16 (21.62%)	0.079
Dyspnea, n (%)	43 (15.52%)	30 (14.78%)	13 (17.57%)	0.571
**Comorbidities on admission**				
COPD (%)	1 (0.36%)	1 (0.49%)	0(0%)	0.598
Diabetes Mellitus (%)	27 (9.75%)	21 (10.34%)	6 (8.11%)	0.578
Hypertension (%)	84 (30.32%)	58 (28.57%)	26 (35.14%)	0.293
Chronic liver disease, n (%)	9 (3.25%)	7 (3.45%)	2 (2.7%)	0.941
Chronic renal diseases, n (%)	6 (2.17%)	5 (2.46%)	1 (1.35%)	0.924

*P-values were generated by the comparison between Non-antitumor group and Anti-tumor group*.

Regarding treatment, patients in the anti-tumor therapy group experienced a higher accuracy rate of ALI (21.62 vs. 10.34%; *p* = 0.015) compared to the non-anti-tumor therapy group. The accuracy rate of sepsis and septic shock, ARDS, AKI, AMI, and DIC were similar between the two groups. Treatments (antibiotics, antiviral drugs, Chinese patent medicine, vasoactive drugs, hormone therapy drugs, ICU treatment, non-invasive ventilation, invasive ventilation) had no statistical difference between the two groups (Table 4). Clinical end point showed no statistical difference in the mortality rate for the two groups (anti-tumor therapy group, 9.46% vs. non-anti-tumor therapy group, 8.87%, *p* = 0.879; Table 4).

**Table 4:** Treatment and Outcome of patients with COVID-19 in Non-antitumor group and Antitumor group.

**Parameters**	**Total population**	**Non- antitumor group**	**Antitumor group**	***P***
	***N* = 277**	***N* = 203**	***N* = 74**	
	**Median (range) or n (%)**	**Median (range) or n (%)**	**Median (range) or n (%)**	
**Treatment**				
Antiviral drug, n (%)	207 (74.73%)	147 (72.41%)	60 (81.08%)	0.142
Antibiotics, n (%)	199 (71.84%)	144 (70.94%)	55 (74.32%)	0.579
Traditional Chinese medicine, n (%)	218 (78.70%)	162 (79.80%)	56 (75.68%)	0.458
Antidiabetic drug, n (%)	58 (20.94%)	42 (20.69%)	16 (21.62%)	0.866
Vasoactive drug, n (%)	43 (15.52%)	31 (15.27%)	12 (16.22%)	0.848
Systemic corticosteroids, n (%)	97 (35.02%)	66 (32.51%)	31 (41.89%)	0.148
Immunoglobin, n (%)	76 (27.44%)	54 (26.60%)	22 (29.73%)	0.606
Nasal cannula oxygen inhalationd, n (%)	224 (80.87%)	166 (81.77%)	58 (78.38%)	0.525
Invasive ventilation, n (%)	17 (6.14%)	12 (5.91%)	5 (6.76%)	0.981
Noninvasive ventilation, n (%)	27 (9.75%)	19 (9.36%)	8 (10.81%)	0.719
Renal replacement therapy, n (%)	3 (1.08%)	2 (0.99%)	1 (1.35%)	0.692
Extracorporeal membrane oxygenation, n (%)	1 (0.36%)	1 (0.49%)	0 (0%)	0.598
**Outcome**				
Sepsis and Septic shock, n (%)	20 (7.22%)	13 (6.40%)	7 (9.46%)	0.385
ARDS, n (%)	50 (18.05%)	33 (16.26%)	17 (22.97%)	0.198
Acute kidney injury, n (%)	20 (7.22%)	14 (6.90%)	6 (8.11%)	0.730
Acute liver injury, n (%)	37 (13.36%)	21 (10.34%)	16 (21.62%)	0.015^*^
Acute myocardial injury, n (%)	35 (12.64%)	26 (12.81%)	9 (12.16%)	0.886
DIC, n (%)	7 (2.53%)	5 (2.46%)	2 (2.70%)	0.748
ICU stay, n (%)	30 (10.83%)	22 (10.84%)	8 (10.81%)	0.995
Mortality, n (%)	25 (9.03%)	18 (8.87%)	7 (9.46%)	0.879

*P-values were generated by the comparison between Non-antitumor group and Anti-tumor group*,

* *P < 0.05*.

## Discussion

In the present study, we reported a considerably high prevalence of COVID in tumor patients (277/7, 926, 3.49%). We speculate the reason is two-fold. First, tumor patients were highly exposed to the risk of virus infection, because they require more hospitalization or outpatient visits. Also, the immune homeostasis of the tumor patients was blunted (16, 17), especially those receiving chemotherapy, immunosuppressive therapy or molecular-targeted drug therapy, even those newly diagnosed patients have not been subjected to treatment or patients with best supportive care, their immune status were compromised (18).

Findings from this study indicated that cancer patients are characterized by older age, but lower prevalence of cough and higher prevalence of fever at onset stage. Of note, the duration between onset and admission was shorter in the cancer group than in the non-cancer group. This may be attributed to the blunted immune status in cancer patients. Previous studies have demonstrated that cancer patients have a higher risk of severe events (19). This study reported that the physiological severity of illness such as sepsis and septic shock, ARDS, AKI, ALI, AMI, and DIC occurred frequently in cancer patients. Besides, they also experienced more antiviral drugs, antibiotics, vasoactive drugs, hormone therapy drugs, ICU treatment, non-invasive ventilation and invasive ventilation. This indicates the health care providers more resources such as medications, medical supplies, modern equipments, employees were needed to allocate to the population. Even though, higher mortality occurred in the patients of cancer group. These findings remind us, to decrease the incidence of virus infection, phone communication or network consulting service must be considered by both physicians and patients as a way to improve care and follow-up and to reduce unnecessary visits to hospitals during the outbreak (20–22). Urgent and semi-urgent patients who require hospital treatment or check-up should do more protections to prevent nosocomial infection.

Several oncology societies have developed guidelines on cancer care during COVID-19 pandemic. However, there are some questions that remain open, including the risk of impairing the outcome when treatment stopped, continued, or modified for the patient's well-being and the “distraction effect” of the pandemic, which is represented by the risk of shifting total attention away from standard clinical care to COVID-19 only (23). ESMO, NICE and French guidelines suggested to use a tiered approach to categorize patients into different priority levels to receive active cancer therapy (24). This study reports that patients who underwent anti-tumor therapy in the past 6 month exhibited a higher accuracy rate of ALI. In the previse study, *Yekedüz E* reported chemotherapy increased the risk of death from COVID-19 in cancer patients, but there was no safety concern for immunotherapy, targeted therapies, surgery and radiotherapy (25). *Song K* demonstrated a possible association between recent receipt of oncologic treatment and a higher risk of death among patients with carcinoma who are hospitalized with COVID-19 (26). However, the findings of the current study do not support the previous research (19, 25). One unanticipated result was that the risk accuracy rate for severe events such as sepsis and septic shock, ARDS, AKI, AMI, and DIC were similar in the anti-tumor group compared with patients not subjected to anti-tumor therapy in the past 6 months. Interestingly, treatments of both populations experienced also had no statistical difference. Anti-tumor therapy (chemotherapy, radiotherapy, targeted therapy) in the past 6 months did not affect the mortality of cancer patients.

A note of caution is due here since a substantial proportion of patients who have longer disease courses may be clinically cured, or those new discovered patients incidentally during COVID-19 therapy may be mixed in the group not subjected to anti-tumor therapy. Even though, it is particularly encouraging to find that the patients who underwent antitumor treatments (chemotherapy, radiotherapy, targeted therapy) in the past 6-month still showed no worse outcome compared with the other group. The results of our study are similar to the results of the study conducted by *Jee J* et al. In their study, patients treated with cytotoxic chemotherapy did not have an increased risk of worse COVID-19 course (27). Interestingly, *K Yang* reported receiving chemotherapy within 4 weeks before symptom onset, and male sex were risk factors for death during admission to hospital (8). However, the effect of treatment on its postponement of cancer patients cannot be ignored (28, 29), the diagnostic or treatments' delays would result in life-years lost. We proposed that streamlined efficient anti-tumor treatment should not be affected or cancelled. Originally prescribed antitumor regimens are recommended when sufficient resources and standard precautions can be ensured.

However,there are still some limitations in our study and we have a lot of works to do in the future. First, patients enrolled were infected with early variants of the virus during the time frame of the first outbreak. The effect to cancer patients might change accompanied by viral mutations. Subsequent variants which might potentially result to a slightly different patient presentation. Second, our main aim was to check whether anti-tumor therapy in the past 6 months could worsen the prognosis of cancer patients with COVID-19. Treatment and outcome of different types of cancer with COVID-19 in non-antitumor group and antitumor group were illustrated. However, the older age for the cancer group might be biased by the types of cancer detected, especially as older patients might have a greater proportion of comorbidities. Limitations in sample size have hampered a more stratified analysis to reduce the effect of selection bias. In addition, we only conducted analysis and research in cohorts in Hubei, China. For patients in other regions, the conclusions we observed may be subject to geographic influences, including local medical conditions, economic levels, and government policies.

Although the current study enrolled limited patients, the findings confirmed the vulnerability of cancer patients with the SARS-CoV-2 (30). Moreover, we suggest that tumor patients should be considered as a special population because they are more susceptible to infection with SARS-CoV-2 due to immune change and social medical need, Antitumor treatments do not expose tumor patients to more risk of severe complications or higher mortality when they have SARS-CoV-2 infection. The antitumor treatments for tumor patients during pandemic should be recommended cautiously.

## Data Availability Statement

The raw data supporting the conclusions of this article will be made available by the authors. Meanwhile, the proposal with detailed aims, statistical plan, and other information/materials may be required and investigated by the 19 hospitals to guarantee the rationality of requirement and the security of the data.

## Ethics Statement

The studies involving human participants were reviewed and approved by Central Ethics Committees and Institutional Ethics Committees in Zhongnan Hospital. Written informed consent for participation was not required for this study in accordance with the national legislation and the institutional requirements.

## Author Contributions

HW and YY: conception and design and acquisition of data (acquired and managed patients, provided facilities, etc.). DG and HW: development of methodology. DG, HW, and QZ: analysis and interpretation of data (e.g., statistical analysis, biostatistics, computational analysis), writing, review, and revision of the manuscript. DG and YY: administrative, technical, and material support (i.e., reporting or organizing data, constructing databases). YY: study supervision. All authors contributed to the article and approved the submitted version.

## Funding

This study was supported by a grant from the National Key Research and Development Program of China (2020YFC0845500 to YY), Zhongnan Hospital of Wuhan University Science, Technology and Innovation Seed Fund (CXPY2020015) and Cancer research and translational platform project of Zhongnan Hospital of Wuhan University (ZLYNXM202004).

## Conflict of Interest

The authors declare that the research was conducted in the absence of any commercial or financial relationships that could be construed as a potential conflict of interest.

## Publisher's Note

All claims expressed in this article are solely those of the authors and do not necessarily represent those of their affiliated organizations, or those of the publisher, the editors and the reviewers. Any product that may be evaluated in this article, or claim that may be made by its manufacturer, is not guaranteed or endorsed by the publisher.

## Abbreviations

AIDS: acquired immune deficiency syndrome
AKI: acute kidney injury
ALI: acute liver injury
ALP: alkaline phosphatase
ALT: alanine aminotransferase
AMI: acute myocardial injury
ARDS: acute respiratory distress syndrome
AST: aspartate aminotransferase
CI: confidence interval
COPD: chronic obstructive pulmonary disease
CoV-2: coronavirus 2
COVID-19: coronavirus disease 2019
CRP: C-reactive protein
CT: computed tomography
DIC: disseminated intravascular coagulation
ECMO: extracorporeal membrane oxygenation
IQR: interquartile range
PCT: procalcitonin
SARS: severe acute respiratory syndrome
TBIL: total bilirubin
ULN: upper limit of normal.
