# Supplementary material for: Clinical Characteristics of Cancer Patients With COVID-19: A Retrospective Multicentric Study in 19 Hospitals Within Hubei, China
Source: Front Med (Lausanne). 2021 Oct 5;8:614057. doi: 10.3389/fmed.2021.614057 (PMC8523781; doi:10.3389/fmed.2021.614057)
Supplement: Supplementary file 1 [file Table_1.docx]

| **Table S1 Chest CT and Laboratory examination of patients with COVID-19 in Non-cancer group and cancer group on admission**. | | | | | | | | |
| --- | --- | --- | --- | --- | --- | --- | --- | --- |
| **Parameters** | **Total population** | | **Non-cancer group** | | **Cancer group** | | ***P*** |  |
|  | N=7926  median(range) or n(%) | | N=7649  median(range) or n(%) | | N=277  median(range) or n(%) | |  |  |
| **Chest CT on admission** |  |  |  |  |  |  |  |  |
| No lesion, n/N (%) | 1080(13.62%) | | 1066(13.94%) | | 14(5.05%) | | <0.001** |  |
| Unilateral lesion, n/N(%) | 878(11.08%) | | 844(11.03%) | | 34(12.27%) | |  |  |
| Bilateral lesions, n/N (%) | 5968(75.30%) | | 5739(75.03%) | | 229(82.67%) | |  |  |
| **Laboratory examination on admission** |  |  |  |  |  |  |  |  |
| Leukocyte count , 10^9/L | 5.4(4.21-6.9) | | 5.4(4.22-6.9) | | 5.33(4.06-7.05) | | 0.938 |  |
| Leukocyte count increase, n(%) | 568(7.17%) | | 534(6.98%) | | 34(12.27%) | | <0.001** |  |
| Neutrophils absolute num. increase ,n(%) | 859(10.84%) | | 807(10.55%) | | 52(18.77%) | | <0.001** |  |
| Lymphocyte count,10^9/L, | 1.29(0.89-1.73) | | 1.3(0.89-1.74) | | 1.06(0.72-1.47) | | <0.001** |  |
| Lymphocyte count  decrease, n(%) | 2532(31.95%) | | 2392(31.27%) | | 140(50.54%) | | <0.001** |  |
| C-reactive protein,mg/L | 5.27(2.57-31.48) | | 5.16(2.5-30.78) | | 11.94(4.58-44.58) | | <0.001** |  |
| C-reactive protein increase, n(%) | 2359(29.76%) | | 2253(29.45%) | | 106(38.27%) | | 0.002** |  |
| lymphocyte percentage,% | 25.5(16.8-33.1) | | 25.7(17.1-33.2) | | 22.9(14.9-32.9) | | <0.001** |  |
| Neutrophil percentage,% | 63.3(54.8-74.5) | | 63.1(54.6-74.1) | | 68.9(58.85-79.72) | | <0.001** |  |
| ALT, U/L | 23(15-39) | | 23.1(15-39) | | 21(14-36) | | 0.066 |  |
| ALT increase ,n(%) | 1155(14.57%) | | 1111(14.52%) | | 44(15.88%) | | 0.529 |  |
| AST, U/L | 23(17.45-34) | | 23(17-34) | | 25(19-36) | | 0.023* |  |
| AST increase ,n(%) | 1191(15.03%) | | 1130(14.77%) | | 61(22.02%) | | <0.001** |  |
| ALP, U/L | 64(51-81) | | 64(51-81) | | 69(54.5-92) | | <0.001** |  |
| ALB, g/L | 38.1(34.8-41.2) | | 38.1(34.8-41.3) | | 36.95(33-39.9) | | <0.001** |  |
| GLB, g/L | 27.2(24.3-30.5) | | 27.2(24.4-30.4) | | 27.3(24-31.3) | | 0.550 |  |
| TBIL, μmol/L | 10.3(7.9-13.9) | | 10.3(7.9-13.9) | | 10.45(7.9-15.03) | | 0.295 |  |
| DBIL, μmol/L | 3.1(2.2-4.4) | | 3.04(2.2-4.4) | | 3.3(2.38-5.2) | | 0.007** |  |
| IDBIL, μmol/L | 7(5.1-9.9) | | 7(5.1-9.9) | | 7(4.9-10.28) | | 0.988 |  |
| UREA, mmol/L | 4.4(3.47-5.59) | | 4.39(3.46-5.5) | | 4.84(3.69-6.62) | | <0.001** |  |
| CREA, μmol/L | 63.2(52.1-76.2) | | 63.2(52.1-76.1) | | 62(50.6-80.5) | | 0.962 |  |
| CREA increase ,n(%) | 445(5.61%) | | 415(5.43%) | | 30(10.83%) | | <0.001** |  |
| CK-MB, U/L | 8(1.4-13) | | 8(1.42-13) | | 8(1.19-13) | | 0.576 |  |
| Procalcitonin level increase ,n(%) | 1313(16.57%) | | 1236(16.16%) | | 77(27.80%) | | <0.001** |  |
| P-values were generated by the comparison between Non-cancer group and Cancer group, *P < 0.05, **P < 0.01 | | | | | | | | |
|  | | | | | | | | |
