# Supplementary material for: Clinical Characteristics of Cancer Patients With COVID-19: A Retrospective Multicentric Study in 19 Hospitals Within Hubei, China
Source: Front Med (Lausanne). 2021 Oct 5;8:614057. doi: 10.3389/fmed.2021.614057 (PMC8523781; doi:10.3389/fmed.2021.614057)
Supplement: Supplementary file 2 [file Table_2.docx]

| **Table S2 Chest CT and Laboratory examination of cancer patients with COVID-19 in Non-antitumor group and Antitumor group on admission.** | | | | | | | |
| --- | --- | --- | --- | --- | --- | --- | --- |
| **Parameters** | **Total population** | | **Non-** **antitumor group** | | **Antitumor group** | | ***P*** |
|  | N=277  median(range) or n(%) | | N=203  median(range) or n(%) | | N=74  median(range) or n(%) | |  |
| **Chest CT on admission** |  |  |  |  |  |  |  |
| No lesion, n/N (%) | 14(5.05%) | | 9(4.43%) | | 5(6.75%) | | 0.791 |
| Unilateral lesion, n/N(%) | 34(12.27%) | | 24(11.82%) | | 10(13.51%) | |  |
| Bilateral lesions, n/N (%) | 229(82.67%) | | 166(81.77%) | | 63(85.13%) | |  |
| **Laboratory examination on admission** |  |  |  |  |  |  |  |
| Leukocyte count , 10^9/L | 5.33(4.06-7.05) | | 5.29(4.02-6.8) | | 5.66(4.34-8.36) | | 0.177 |
| Leukocyte count increase, n(%) | 34(12.27%) | | 21(10.34%) | | 13(17.57%) | | 0.105 |
| Neutrophils absolute num. increase ,n(%) | 52(18.77%) | | 32(15.76%) | | 20(27.03%) | | 0.034* |
| Lymphocyte count,10^9/L, | 1.06(0.72-1.47) | | 1.02(0.68-1.46) | | 1.12 (0.83-1.53) | | 0.685 |
| Lymphocyte count decrease, n(%) | 140(50.54%) | | 102(50.25%) | | 38(51.35%) | | 0.871 |
| C-reactive protein, mg/L | 11.94(4.58-44.58) | | 8(1.84-45.9) | | 12.55(4.68-30.37) | | 0.106 |
| C-reactive protein increase, n(%) | 106(38.27%) | | 74(36.45%) | | 32(43.24%) | | 0.304 |
| lymphocyte percentage,% | 22.9(14.9-32.9) | | 23.7(15-33.3) | | 22.45(13.9-31.55) | | 0.445 |
| Neutrophil percentage,% | 68.9(58.85-79.72) | | 68.8(58.8-79.8) | | 68.65(58.17-76.12) | | 0.777 |
| ALT, U/L | 21(14-36) | | 23(15.7-38.2) | | 19(13-35) | | 0.546 |
| ALT increase ,n(%) | 44(15.88%) | | 30(14.78%) | | 14(18.92%) | | 0.404 |
| AST, U/L | 25(19-36) | | 25(17-35) | | 26(19-40) | | 0.261 |
| AST increase ,n(%) | 61(22.02%) | | 40(19.70%) | | 21(28.38%) | | 0.123 |
| ALP, U/L | 69(54.5-92) | | 75(51-94) | | 68(61.25-87.75) | | 0.378 |
| ALB, g/L | 36.95(33-39.9) | | 37.2(33.6-40.1) | | 36.6(32.9-39.6) | | 0.609 |
| GLB, g/L | 27.3(24-30.3) | | 27.6(24.67-30.83) | | 25.25(22.15-27.83) | | <0.001** |
| TBIL, μmol/L | 10.45(7.9-15.03) | | 10.35(8.0-15.1) | | 11.5(7.9-14.78) | | 0.266 |
| DBIL, μmol/L | 3.3(2.38-5.2) | | 3.2(2.35-4.8) | | 3.5(2.43-4.95) | | 0.579 |
| IDBIL, μmol/L | 7(4.9-10.28) | | 6.9(4.75-9.1) | | 7.9(5.08-10.47) | | 0.696 |
| UREA, mmol/L | 4.84(3.69-6.62) | | 4.66(3.37-6.3) | | 5.2(3.73-6.96) | | 0.083 |
| CREA, μmol/L | 62(50.6-80.5)) | | 61.9(51-79.2) | | 64(49.75-83) | | 0.804 |
| CREA increase ,n(%) | 30(10.83%) | | 20(9.85%) | | 10(13.51%) | | 0.386 |
| CK-MB, U/L | 8(1.19-13) | | 8.2(1.14-15) | | 7(2-12.1) | | 0.999 |
| Procalcitonin level increase ,n(%) | 77(27.80%) | | 60(29.56%) | | 17(22.97%) | | 0.279 |
| P-values were generated by the comparison between Non-antitumor group and Antitumor group, *P < 0.05, **P < 0.01 | | | | | | | |
|  | | | | | | | |
