# Supplementary material for: Clinical Characteristics of Cancer Patients With COVID-19: A Retrospective Multicentric Study in 19 Hospitals Within Hubei, China
Source: Front Med (Lausanne). 2021 Oct 5;8:614057. doi: 10.3389/fmed.2021.614057 (PMC8523781; doi:10.3389/fmed.2021.614057)
Supplement: Supplementary file 3 [file Table_3.docx]

| **Table S3 Treatment and Outcome of patients with COVID-19 in Non-antitumor group and Antitumor group.** | | | | | |
| --- | --- | --- | --- | --- | --- |
| **Site** | **Total(deaths)** | **percentage** | **anti-tumor**  **(deaths)** | **percentage** | ***P*** |
| Lip, oral cavity, & pharynx (except nasopharynx) | 1 | 0.36% | 1 | 1.35% |  |
| Nasopharynx | 4 | 1.44% | 2 | 2.70% |  |
| Esophagus | 9 | 3.25% | 2 | 2.70% |  |
| Stomach | 18(2) | 6.50% | 6(1) | 8.11% | 0.790 |
| Colorectum | 30(3) | 10.83% | 7(1) | 9.46% | 0.773 |
| Liver | 9 | 3.25% | 1 | 1.35% |  |
| Gallbladder | 0 | 0.00% | 0 | 0.00% |  |
| Pancreas | 7(1) | 2.53% | 2 | 2.70% | 0.608 |
| Larynx | 2 | 0.72% | 0 | 0.00% |  |
| Lung | 50(11) | 18.05% | 18(4) | 24.32% | 0.743 |
| Other thoracic organs | 2 | 0.72% | 0 | 0.00% |  |
| Bone | 6 | 2.17% | 3 | 4.05% |  |
| Melanoma of the skin | 3 | 1.08% | 3 | 4.05% |  |
| Breast | 39(2) | 14.08% | 14(1) | 18.92% | 0.741 |
| Cervix | 8(1) | 2.89% | 2 | 2.70% | 0.537 |
| Uterus | 3 | 1.08% | 1 | 1.35% |  |
| Ovary | 7 | 2.53% | 2 | 2.70% |  |
| Prostate | 2 | 0.72% | 1 | 1.35% |  |
| Testis | 0 | 0.00% | 0 | 0.00% |  |
| Kidney | 10 | 3.61% | 1 | 1.35% |  |
| Bladder | 12(1) | 4.33% | 2 | 2.70% | 0.350 |
| Brain, CNS | 11(1) | 3.97% | 2 | 2.70% | 0.387 |
| Thyroid | 23(1) | 8.30% | 0 | 0.00% | - |
| Lymphoma | 2 | 0.72% | 0 | 0.00% |  |
| Leukemia | 15(2) | 5.42% | 3 | 4.05% | 0.849 |
| All other sites and unspecified | 4 | 1.44% | 1 | 1.35% |  |
| Total | 277(25) | 100 | 74(7) | 100 | 0.879 |
| P-values were generated by the comparison of deaths between Non-antitumor group and Anti-tumor group. | | | | | |
