# Supplementary material for: Clinical Characteristics of Cancer Patients With COVID-19: A Retrospective Multicentric Study in 19 Hospitals Within Hubei, China
Source: Front Med (Lausanne). 2021 Oct 5;8:614057. doi: 10.3389/fmed.2021.614057 (PMC8523781; doi:10.3389/fmed.2021.614057)
Supplement: Supplementary file 4 [file Table_4.docx]

**Table S4 Criteria definition used in the research.**

| **Parameters** | **Criteria definition** |
| --- | --- |
| Acute kidney injury | An elevation in serum creatinine level equal or above 26.5 mmol/L within 48 hours |
| Acute cardiac injury | Serum level of cardiac troponin I (cTNI), cardiac troponin T (cTNT), or high sensitivity cardiac troponin I (hs-cTNI) above the upper limit of normal (ULN) |
| Acute liver injury | Patients who had at least one condition of the follows: 1)raised ALT and/or AST more than 3 times the upper limit units (ULN); 2)raised ALP or GGT twice the ULN; 3) raised combination of both ALT/AST more 3 time the ULN and ALP/GGT twice the ULN |
| Disseminated intravascular coagulation (DIC) | International Society on Thrombosis and Hemostasis (ISTH) |
| Increase in blood cell count or biochemical indexes | Over their ULN according to the criteria by the laboratory standards in each hospital |
| Sepsis and septic shock | Clinical management of severe acute respiratory infection when novel coronavirus (2019-nCoV) infection is suspected |
| ARDS | Clinical management of severe acute respiratory infection when novel coronavirus (2019-nCoV) infection is suspected |
|  |  |
